# Cancer survival prediction by learning comprehensive deep feature representation for multiple types of genetic data

**DOI:** 10.1186/s12859-023-05392-z

**Published:** 2023-06-28

**Authors:** Yaru Hao, Xiao-Yuan Jing, Qixing Sun

**Affiliations:** 1 School of Computer Science, Wuhan University, Wuhan, China; 2 School of Computer, Guangdong University of Petrochemical Technology, Maoming, China; 3 State Key Laboratory for Novel Software Technology, Nanjing University, Nanjing, China

**Keywords:** Cancer survival prediction, Shared information, Specific information, Comprehensive representation

## Abstract

**Background:**

Cancer is one of the leading death causes around the world. Accurate prediction of its survival time is significant, which can help clinicians make appropriate therapeutic schemes. Cancer data can be characterized by varied molecular features, clinical behaviors and morphological appearances. However, the cancer heterogeneity problem usually makes patient samples with different risks (i.e., short and long survival time) inseparable, thereby causing unsatisfactory prediction results. Clinical studies have shown that genetic data tends to contain more molecular biomarkers associated with cancer, and hence integrating multi-type genetic data may be a feasible way to deal with cancer heterogeneity. Although multi-type gene data have been used in the existing work, how to learn more effective features for cancer survival prediction has not been well studied.

**Results:**

To this end, we propose a deep learning approach to reduce the negative impact of cancer heterogeneity and improve the cancer survival prediction effect. It represents each type of genetic data as the shared and specific features, which can capture the consensus and complementary information among all types of data. We collect mRNA expression, DNA methylation and microRNA expression data for four cancers to conduct experiments.

**Conclusions:**

Experimental results demonstrate that our approach substantially outperforms established integrative methods and is effective for cancer survival prediction.

**Availability and implementation:**

https://github.com/githyr/ComprehensiveSurvival.

## Introduction

As the morbidity and mortality rates gradually rise, cancer is becoming the main death cause in the global [1–3]. According to the global cancer report, additional 14.10 million cancer cases occurred with death cases 8.20 million in 2012. The number of new cancer cases and death cases reached 18.1 million (9.5 million men and 8.6 million women) and 9.6 million respectively in 2018 [4]. Meanwhile, cancers have become common among young people. Therefore, it is significant to accurately predict the survival time, which can help the clinicians make proper therapeutic guidance to improve the survival rate and living quality of cancer patients [5, 6].

**Fig. 1:**
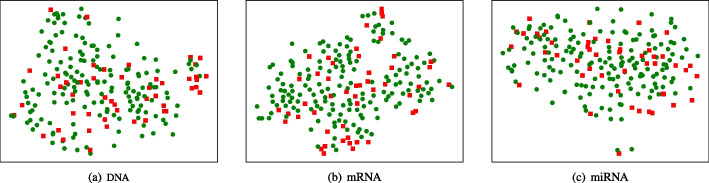
Visualizations of embedding feature spaces of DNA methylation, mRNA expression and microRNA expression for GBM. The red dots point short time survivors (< 2 years)and the blue dots represent long time survivors (> 2 years)

Cancer survival prediction has been an interesting and challenging issue in cancer research over the past few decades [7–9]. The heterogenous disease, cancer, can be characterized by varied molecular features, clinical behaviors, morphological appearances and reactions to therapies. This leads that the genes and phenotypes of cells in the same pattern and stage are also different, which results in a big challenge for cancer survival prediction [10–12]. Figure 1 visualizes the embedding feature spaces of DNA methylation, mRNA expression and microRNA expression for the glioblastoma multiforme (GBM) dataset by reducing the dimensionality of original features. Specifically, we use a commonly-used visualization method t-SNE (t-distributed stochastic neighbor embedding) [13] to display the low-dimensional feature space of genetic data. t-SNE adopts the nonlinear dimensionality reduction technique and can preserve the local and global distribution structures of the dataset. As shown in this figure, patient samples with different survival times are mixed together and difficult to be distinguished in the embedding feature spaces of three types of genetic data, which further verifies the difficulty of cancer survival prediction.

Survival analysis is usually accomplished using heterogeneous data sources including low-dimensional clinical data (age, sex, cancer grade detail, body fat rate, etc.) [14], pathological images [15–18], and multi-type gene data [19]. For example, Chen et al. proposed an interpretable strategy for end-to-end multimodal fusion of histology image and genomic (mutations, CNV, RNA-Seq) features [20]. Cheerla et al. designed an unsupervised encoder to integrate four data modalities (gene expression data, miRNA data, clinical data and whole slide image) into a single feature vector for each patient [21]. Vale-Silva et al. utilized clinical, imaging, and different high-dimensional omics data modalities to conduct cancer survival prediction [22]. Compared with single source data, multi-source heterogeneous data describes the cancer from different perspectives, which can capture a more comprehensive understanding of the cancer [23, 24]. Multi-source heterogeneous data can be regarded as multi-modal data, which contains not only large consensus information but also abundant complementary information [25]. From information perspective, consensus information indicates that each modality contains information that shared by all modalities (inter-modal shared information); complementary information instructs that each modality also contains information that is unique to itself (intra-modal specific information) [26].

**Fig. 2:**
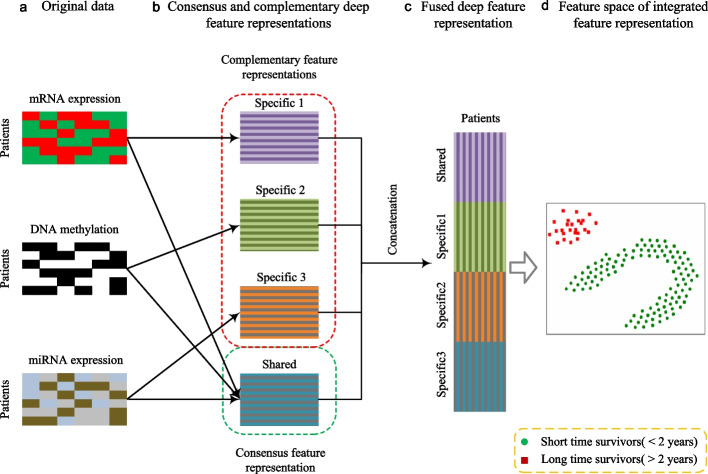
A demo for deep learning for cancer survival prediction. **a** Example representations of mRNA expression, DNA methylation and miRNA expression datasets for the same cohort of patients. **b** Learning deep feature representation from the perspective of shared and specific information. **c** Fused deep feature representation by concatenation for cancer survival prediction. **d** The visualization of embedding feature space of integrated feature representation

Existing data integration based cancer survival prediction methods can be mainly categorized into three paradigms:

(i) fusion methods [27–30] based on concatenation integrate multiple types of data directly. This seems unreasonable since the concatenation of heterogeneous data sources neglects the inter-modal discriminant information. In addition, this strategy would cause very high dimensional feature vectors, which is adverse for feature learning [31].
(ii) fusion methods [21, 32–34] only learn consensus information. This strategy only exploits the consensus information of heterogeneous data but ignores the diversity of heterogeneous data, which is adverse for exploiting comprehensive information of cancer. While, for heterogenous disease, making full use of the complementarity between different types of data is conducive to a comprehensive understanding of the disease.
(iii) works [25] and [35] utilize the similarity network fusion to integrate multiple types of data. They learn consensus and complementary information based on the relations between patient samples. However, they ignore fine-grained feature representation information, especially for gene sequences with thousands of dimensions.

In general, although multi-type gene data have been used in the existing work, how to learn more effective features for cancer survival prediction and explain them at the feature level has not been well studied. Besides, deep learning technique has been proved to have strong feature representation and classification ability in various tasks. In this paper, we intend to utilize deep learning to obtain more effective feature representations of multi-type genetic data and achieve better performance of cancer survival prediction at the feature level, which can reduce the negative impact of data heterogeneity. Also, we want to explain the functions of deep features from the aspect of extracting consensus and complementary information. Figure 2 shows a demo of the proposed deep learning for cancer survival prediction. These consensus and complementary representations are exploited to capture comprehensive survival information of cancer patients; e.g., consensus representation is exploited to capture modality-invariant survival information; the specific representations of mRNA expression, DNA methylation and miRNA expression are exploited to capture the modality-specific survival information.

The main contributions of this study are summarized as follows:

1. This study focuses on the problem of data heterogeneity in cancer survival prediction and proposes a deep learning approach to integrate multi-type genetic data effectively. As shown in Fig. 2, by sufficiently integrating multi-type genetic data, survivors with different times (i.e., short and long times) can be well separated in the feature space built by our approach, which means that the negative impact of data heterogeneity on cancer survival prediction can be alleviated significantly.
2. In the proposed deep learning approach, it represents each type of genetic data as the shared and specific features, which can capture the consensus and complementary information among all types of data. Then, we fuse the shared and specific features of each type of data by concatenation, and employ the fused features for cancer survival prediction as shown in Fig. 2. To strengthen the representation ability of deep features, we layer-by-layer impose an Euclidean distance constraint on the shared feature learning network, as well as impose an orthogonal constraint on the specific feature learning network.
3. We conduct extensive experiments on glioblastoma multiforme (GBM), kidney renal clear cell carcinoma (KRCCC), lung squamous cell carcinoma (LSCC) and breast invasive carcinoma (BIC) datasets. Experimental results show that our approach can achieve higher prediction performance than competing methods. This demonstrates that our approach significantly improves the performance of cancer survival prediction and is helpful for clinicians to make proper therapeutic guidance for cancer patients.

## Proposed methods

**Fig. 3:**
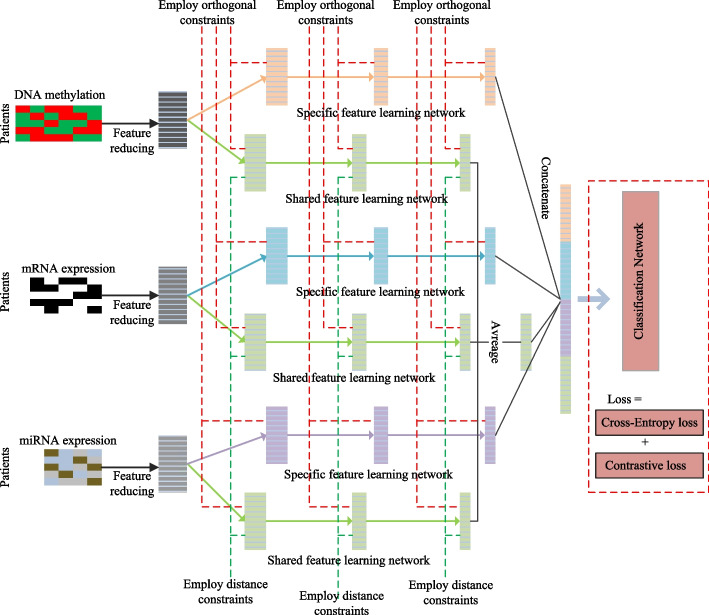
The architecture of our proposed deep learning approach

Figure 3 shows the proposed deep learning network to achieve the shared and specific feature representation for cancer survival prediction. First, it maps the original feature dimensions of all data types to the same dimension through a fully connected neural network. Secondly, it builds a multi-stream deep shared network with parameters shared for all data types to learn the consensus information, as well as a deep specific network for each data type to learn the complementary information. At the same time, an Euclidean distance constraint is used to enhance the learning of consensus information and an orthogonal constraint is used to enhance the learning of complementary information. Finally, to improve the separability of data, we introduce the contrastive loss to pull samples from the same class closer and push samples from different classes farther.

### Feature mapping

The data integration strategy designed in this paper can learn the consensus and complementary information only when the feature dimensions of these data types are consistent. Therefore, it is necessary to map the features of all genetic data types to the same dimension. A common approach is to adopt the Max-Relevance and Min-Redundancy (mRMR) feature selection algorithm for dimension reduction [27, 36, 37], which ignores the interaction between gene sites in sequence. In this paper, we design a three-layer fully connected neural network for feature mapping. Considering that the dimension increase operation will introduce noise, we employ dimension reduction operation to get the same feature dimension for all data types. In addition, the data dimension for miRAN is relatively low (329 dimension for KRCCC to 534 dimension for GBM), which is not suitable for further dimensionality reduction. Therefore, we use the dimension of miRNA as the last mapping dimension. Table 1 shows the detailed dimensionality values for the feature mapping process.

**Table 1:** Feature mapping process of three data types for four cancer datasets

Datasets	Modality	Dimensionality	The dimension of ANN	Last mapping dimension
GBM	mRNA	12042	12042$\rightarrow$4096$\rightarrow$534	534
	miRNA	534	534$\rightarrow$534$\rightarrow$534	534
	DNA	1305	1305$\rightarrow$768$\rightarrow$534	534
KRCCC	mRNA	17899	17899$\rightarrow$4096$\rightarrow$329	329
	miRNA	329	329$\rightarrow$329$\rightarrow$329	329
	DNA	24960	24960$\rightarrow$4096$\rightarrow$329	329
LSCC	mRNA	12042	12042$\rightarrow$4096$\rightarrow$352	352
	miRNA	352	352$\rightarrow$352$\rightarrow$352	352
	DNA	23074	23074$\rightarrow$4096$\rightarrow$352	352
BIC	mRNA	17814	17814$\rightarrow$4096$\rightarrow$354	354
	miRNA	354	354$\rightarrow$354$\rightarrow$354	354
	DNA	23094	23094$\rightarrow$4096$\rightarrow$354	354

### Shared and specific deep feature learning

Let $X=\left\{ x_{i}\in {\mathbb {R}}^{q} \right\} _{i=1}^{N}$ be a set of *N* samples, where *q* represents dimension of each sample. Moreover, let $X_{K}=\left\{ x_{k,i}\in {\mathbb {R}}^{q^{k}} \right\} _{i=1}^{N}$ denote the feature set of *X* in data type *k*, where ${\mathrm{{x}}_{k,i}}$ is the $k\text{{-th}}$ representation of the ${\mathrm{{x}}_i}$ and ${q^k}$ is the dimension of ${\mathrm{{x}}_i}$. Here, $k = 1,2, \ldots ,K$, where *K* denotes the total number of data types. Generally, the $k\mathrm{{-th}}$ representation ${\mathrm{{x}}_{k,i}}$ and the $l\mathrm{{-th}}$ representation ${\mathrm{{x}}_{l,i}}$ of the ${\mathrm{{x}}_i}$, $k \ne l$, are different, because they are usually from different spaces. Therefore, directly concatenating them may not be physically meaningful, and cannot well utilize the complementary property.

Considering the fact that each data type represents the same object from different point of view, different data types not only contain the specific information but also share common information. For ${\mathrm{{x}}_{k,i}}$, we employ the shared feature learning network to project it to get the consensus information by $h_{k,i}^c = W_k^c{\mathrm{{x}}_{k,i}}$, where $W^{c}\in {\mathbb {R}}^{r^{c}\times q_{k}}$, and employ the specific feature learning network to project it to get the complementary information by $h_{k,i}^s = W_k^s{\mathrm{{x}}_{k,i}}$, where $W_{k}^{s}\in {\mathbb {R}}^{r_{k}^{s}\times q_{k}}$. The learned feature representation of ${\mathrm{{x}}_{k,i}}$ can be written as:

1 $$\begin{aligned} {h_{k,i}} = \left( {\begin{array}{*{20}{c}} {h_{k,i}^s}\\ {h_{k,i}^c} \end{array}} \right) = \left( {\begin{array}{*{20}{c}} {W_k^s}\\ {W_k^c} \end{array}} \right) {\mathrm{{x}}_{k,i}}. \end{aligned}$$

Therefore, the final representation with multiple data types can be denoted as:

2 $$\begin{aligned} \begin{aligned} h_{i}=\left[ h_{1,i}^{s\; \, T},h_{2,i}^{s\; \, T},\ldots ,h_{K,i}^{s\; \, T},h_{1,i}^{c\; \, T},h_{2,i}^{c\; \, T},\ldots ,h_{K,i}^{c\; \, T} \right] ^{T}. \end{aligned} \end{aligned}$$

Since the shared information from different data types is almost the same, it is unnecessary to include all of them in the final representation. Instead, we use the average value:

3 $$\begin{aligned} h_i^c = {W^c}{\mathrm{{x}}_i} \buildrel \Delta \over = \frac{1}{K}\sum \limits _{k = 1}^K {W_k^c{\mathrm{{x}}_{k,i}}}. \end{aligned}$$

Finally, the resulting representation of ${\mathrm{{x}}_i}$ can be written as:

4 $$\begin{aligned} h_{i}=\left[ h_{1,i}^{s\; \, T},h_{2,i}^{s\; \, T},\ldots ,h_{K,i}^{s\; \, T},h_{i}^{c\; \, T} \right] ^{T}. \end{aligned}$$

### Layer-by-layer Euclidean distance and orthogonality constraints

We impose the orthogonality constraint between each layer of shared and specific feature learning networks to separate shared and specific information, as well as prevent them from contaminating each other. Furthermore, we impose the Euclidean distance constraint between each layer of multi-stream shared feature learning networks to ensure the similarity of consensus information. Details are described as follows:

Let $H_k^c\,(m)$ and $H_k^s(m)$ be the outputs of shared and specific networks from layer *m*. Orthogonality loss between $H_k^c(m)$ and $H_k^s(m)$ is defined as:

5 $$\begin{aligned} {L_{diff}} = \left\| {H_k^{c}{{(m)}^\text{T}}} {H_k^s(m)} \right\| _F^2, \end{aligned}$$

where $\left\| \cdot \right\| _{F}^{2}$ is the squared Frobenius norm.

Let $H_k^c(m)$ and $H_l^c(m)$ be the outputs of the same layer for data type *k* and *l* in shared feature learning network, respectively. Euclidean distance loss between $H_k^c(m)$ and $H_l^c(m)$ is defined as:

6 $$\begin{aligned} {L_{diss}} = \frac{1}{{2N}}\sum \limits _{n = 1}^N {d_n^2}, \end{aligned}$$

where ${d_n} = \left\| {h_{k,n}^c} \right. - {\left. {h_{l,n}^c} \right\| ^2}$, and $h_{k,n}^c$ and $h_{l,n}^c$ are shared representation of sample ${{\mathrm{x}}_n}$ in data type *k* and *l*, respectively.

### Classification

After integrating multiple genetic data types into a unified representation, we classify them with a multilayer network. Cross-Entropy loss is used for classification.

To improve the separability of data, contrastive loss is implemented. Specifically, for a pair of samples ${{\mathrm{x}}_i}$ and ${{\mathrm{x}}_j}$, we use ${{\mathrm{h}}_i}$ and ${{\mathrm{h}}_j}$ to represent their features extracted by the feature learning network, respectively. The distance between them is computed as:

7 $$\begin{aligned} d({x_i},{x_j}) = \left\| {{h_i}} \right. - {\left. {{h_j}} \right\| _2}. \end{aligned}$$

Contrastive loss between ${\mathrm{{h}}_i}$ and ${\mathrm{{h}}_j}$ is defined as:

8 $$\begin{aligned} {L_{con}} = \frac{1}{{2N}}\sum \limits _{n = 1}^N {\left[ {{y_n}d_n^2 + (1 - {y_n}){{\max }^2}(Margin - {d_n},0)} \right] }, \end{aligned}$$

where ${{\mathrm{d}}_n}$ is the distance of the ${n^{th}}$ paired samples, *Margin* is a threshold, and ${y_n}$ denotes whether the paired samples are from the same class. If they are from the same class, ${y_n} = 1$, otherwise, ${y_n} = 0$.

## Experiments

### Datasets

Four cancer datasets including glioblastoma multiforme (GBM), kidney renal clear cell carcinoma (KRCCC), lung squamous cell carcinoma (LSCC) and breast invasive carcinoma (BIC) are used to evaluate our approach. For each dataset, we collect three types of genetic data, including DNA methylation, mRNA expression and miRNA expression data. The datasets used in this paper are obtained from http://compbio.cs.toronto.edu/SNF/, which are provided and preprocessed by work [25]. It downloads these data from the TCGA website and performs three steps of preprocessing: sample selection, missing-data imputation and normalization. Detailed preprocessing process is described as follows: (i) if one patient sample has more than 20% missing data in a certain data type, then this sample will be removed; (ii) if a certain gene has more than 20% missing values, then this gene will be filtered, otherwise, the k-nearest interpolation is used for complementing this gene; (iii) the z-score transformation is used for normalizing the data samples.

**Fig. 4:**
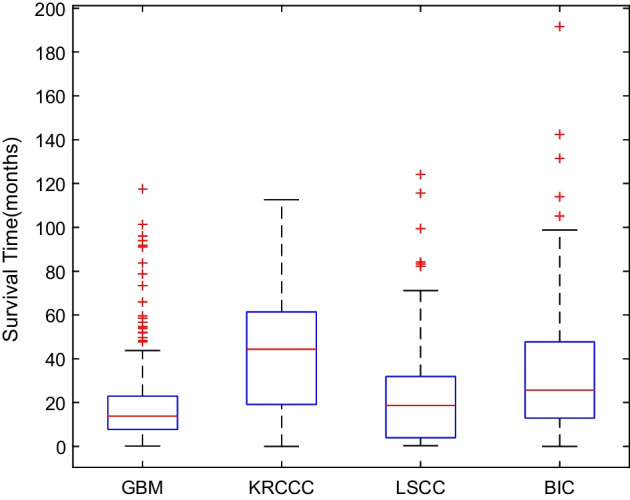
Survival time distribution in four cancer datasets as represented by box plots (center red line represents median, lower and upper quartiles and whiskers capture max and min values of the survival time in each cancer)

Figure 4 illustrates the survival time distribution for four cancer datasets, from which we can observe that the survival time for GBM, KRCCC, LSCC and BIC ranges 0–118 months, 0–113 months, 0–125 months and 0–192 months, respectively. The median survival for GBM, KRCCC, LSCC and BIC is 14 months, 45 months, 19 months and 26 months, respectively. Combined with the survival time distribution and median survival of each cancer, 2-year, 4-year, 2-year and 3-year are taken as thresholds to divide two types of patients with four cancer types. Table 2 shows the data properties of four datasets. For classification, the short term patients are labeled as 0 and long term patients are labeled as 1. The initial feature dimensions of three types of genetic expressions in all datasets are significantly different.

**Table 2:** Data properties of four cancer datasets

Datasets	Instance	Cut-off (years)	Short/long time survivors	Modality	Dimensionality
GBM	215	2	166/49	mRNA	12042
				miRNA	534
				DNA	1305
KRCCC	122	4	67/55	mRNA	17899
				miRNA	329
				DNA	24960
LSCC	106	2	66/40	mRNA	12042
				miRNA	352
				DNA	23074
BIC	105	3	67/38	mRNA	17814
				miRNA	354
				DNA	23094

### Evaluation

To evaluate our proposed method, we adopt ten-fold cross validation in our experiments. Specifically, we randomly divide long time survivors and short time survivors into ten subsets, respectively. For each round of training, each subset of long time survivors combined with each subset of short time survivors will be used as a validation set, another seven subsets of long time survivors combined with seven subsets of short time survivors are used as training set, the last two subsets of long time survivors combined with the last two subsets of short time survivors are used as testing set. The prediction score is the average of the output of ten rounds. In this paper, we use five metrics including Accuracy (*Acc*), Recall, Precision(*Pre*), ROC curve and AUC (area under the ROC curve) to measure model performance. These metrics are defined as follows:

9 $$\begin{aligned} Acc= & {} \frac{{TP + TN}}{{TP + TN + FP + FN}}, \end{aligned}$$

10 $$\begin{aligned} Recall= & {} \frac{{TP}}{{TP + FN}}, \end{aligned}$$

11 $$\begin{aligned} Pre= & {} \frac{{TP}}{{TP + FP}}, \end{aligned}$$

where true positive (TP) represents the number of cases correctly identified as short-survival, false positive (FP) represents the number of cases incorrectly identified as short-survival, true negative (TN) represents the number of cases correctly identified as long-survival, and false negative (FN) represents the number of cases incorrectly identified as long-survival.

### Hyper-parameter selection

The designed cancer survival prediction model consists of three modules: features mapping network, shared and specific representation learning network and classification network. Specifically, the features mapping network adopts a three-layer fully connected network, and the size of each layer is shown in Table 1. We build the shared and specific representation learning network with two hidden fully connected layers of sizes 256 and 128, and an output layer of size 32. Each layer uses the ReLU activation function. The classification network adopts a three-layer fully connected network, in which the sizes of hidden and output layers are 32 and 2, respectively.

To avoid overfitting, we do not perform a separate hyper-parameter search for each cancer dataset. Instead, we search the hyper-parameters on GBM dataset and apply the selected parameters for other datasets. The hyper-parameter margin is searched on the grid [1, 2, 3, 4, 5]. We perform grid search based on the grid [0.0001, 0.0003, 0.0005, 0.0007, 0.0009, 0.001] to determine the learning rate of Adam optimizer. Batch size for training set is searched from [20, 30, 40, 50]. Specifically, we conduct a series of tests on the validation set where in each experiment we vary one of the three hyper-parameters from the chosen value by tuning it up or down by one grid, obtaining 15 sets of varied hyper-parameters. For each set of varied hyper-parameters, 10-fold cross-validation is conducted.

The final chosen hyper-parameters are shown in Table 3.

**Table 3:** The selected hyper-parameters for prediction model

Hyper-parameter	Value
Margin	2.0
Learning rate	0.0003
Batch size	30

### Experimental results

We compare our approach with three state-of-the-art cancer survival prediction methods:

- Similarity network fusion (SNF) for aggregating data types on a genomic scale [25];
- Integrating multiple genomic data and clinical data based on graph convolutional network (GCGCN) for cancer survival prediction [35];
- Multimodal deep neural network for human breast cancer prognosis prediction by integrating multi-dimensional data (MDNNMD) [28].
- Multi-modal advanced deep learning architectures for breast cancer survival prediction (SiGaAtCNNs) [30];
- Cross-aligned multimodal representation learning for cancer survival prediction (CAMR) [38];
- Integrating multi-omics data by learning modality invariant representations for improved prediction of overall survival of cancer (LMIR) [39].

**Table 4:** Brief introduction of our approach and three compared methods

Methods	Deep learning	Data-integration strategies		
		Simple concatenation at feature level	Learning consensus and complementary information at sample level	Consensus and complementary information learning at feature level
SNF	No	No	Yes	No
GCGCN	Yes	No	Yes	No
MDNNMD	Yes	Yes	No	No
SiGaAtCNNs	Yes	Yes	No	No
CAMR	Yes	No	No	Yes
LMIR	Yes	Yes	No	No
Ours	Yes	No	No	Yes

**Fig. 5:**
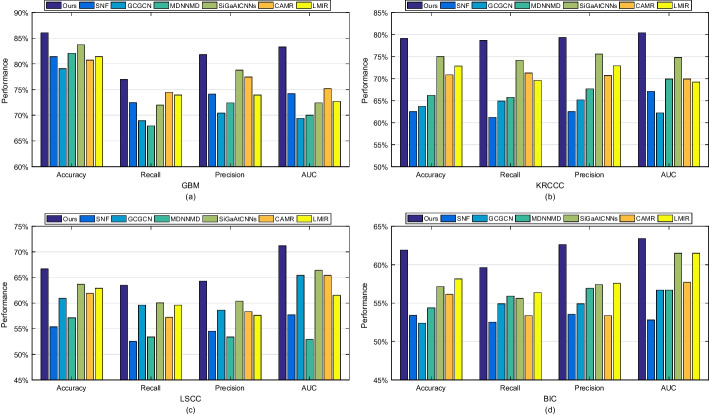
Prediction performance for four cancers survival prediction, comparing SNF, GCGCN, MDNNMD, SiGaAtCNNs, CAMR, LMIR and ours

**Fig. 6:**
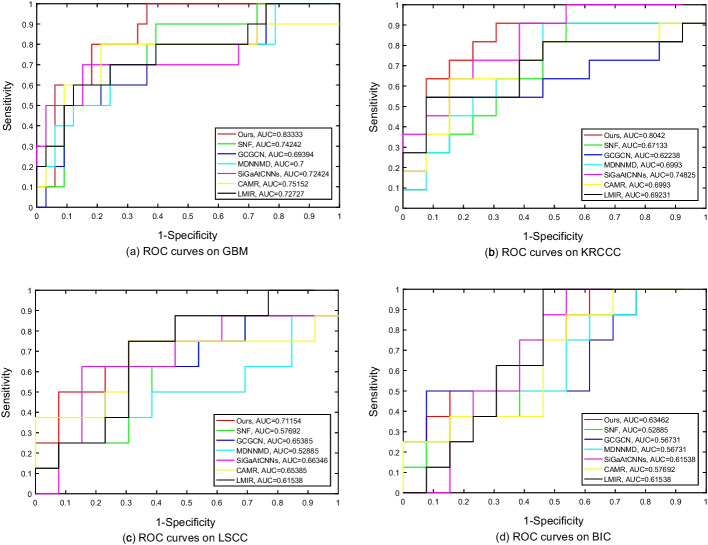
ROC curves for four cancers survival prediction, comparing SNF, GCGCN, MDNNMD, SiGaAtCNNs, CAMR, LMIR and ours

A brief introduction of these survival analysis methods is summarized in Table 4. The predictive results of all competing methods are reported in Figs. 5 and 6. Figure 5 shows the comparison results of all evaluation metrics including accuracy, precision and the area under curve (AUC) on four datasets. Figure 6 presents the receiver operating characteristic (ROC) curves of all competing methods on four datasets.

From these results, we can conclude that the overall performance of our method is much higher than those of three compared methods. This indicates that methods considering consensus and complementary information are better than that simply concatenating features.

**Fig. 7:**
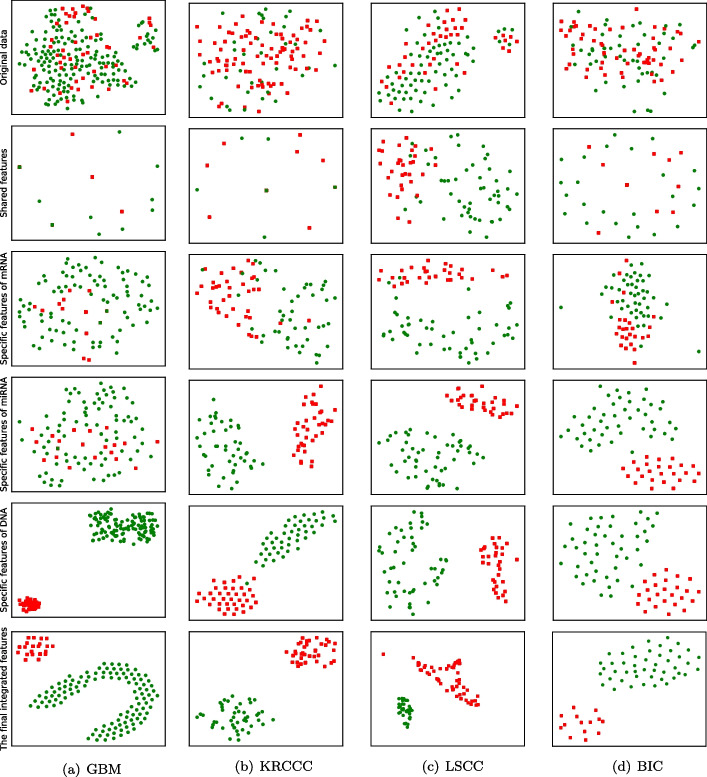
T-SNE visualization of data on each dataset. The top plots in **a**–**d** present the distribution of original samples with concatenated features. The middle four plots in **a**–**d** show the distribution of shared features, specific features of mRNA, specific features of miRNA, and specific features of DNA, respectively. The bottom plots in **a**–**d** show the distribution of samples with learned features by our approach

In order to further investigate the effectiveness of learned feature representations by our approach, i.e., the final fusion representation by concatenating all specific representations and the shared representation, we employ the t-SNE to embed the samples into the two-dimensional space for visualization. Figure 7 illustrates the distribution of original training samples and the distribution of learned feature representations on four cancer datasets. From the figure, we can observe that (1) t-SNE produces visually interpretable results by converting vector similarities into joint probabilities, generating visually distinct clusters that represent patterns in the data. (2) the samples with different survival stages are mixed together and not well separated in the original feature space. (3) With the learned shared features, specific features of mRNA, specific features of miRNA, and specific features of DNA, patients with the same survival stage tend to be clustered. (4) With the final learned integrated features, the samples from different survival stages can be intuitively separated into two disjoint clusters, which indicates the better separability of integrated feature representations.

### Survival analysis

**Fig. 8:**
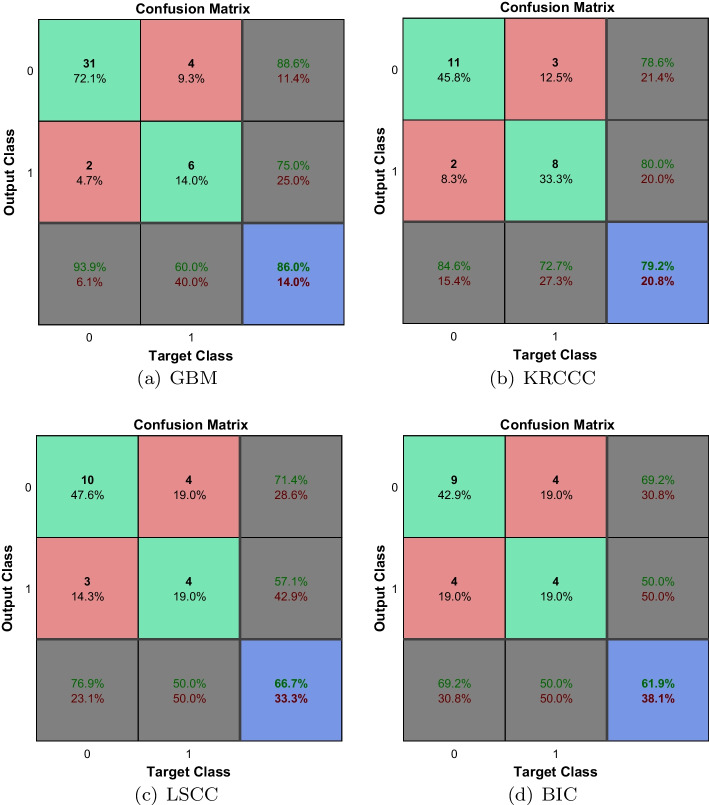
Confusion matrixes of test sets on four cancers

**Fig. 9:**
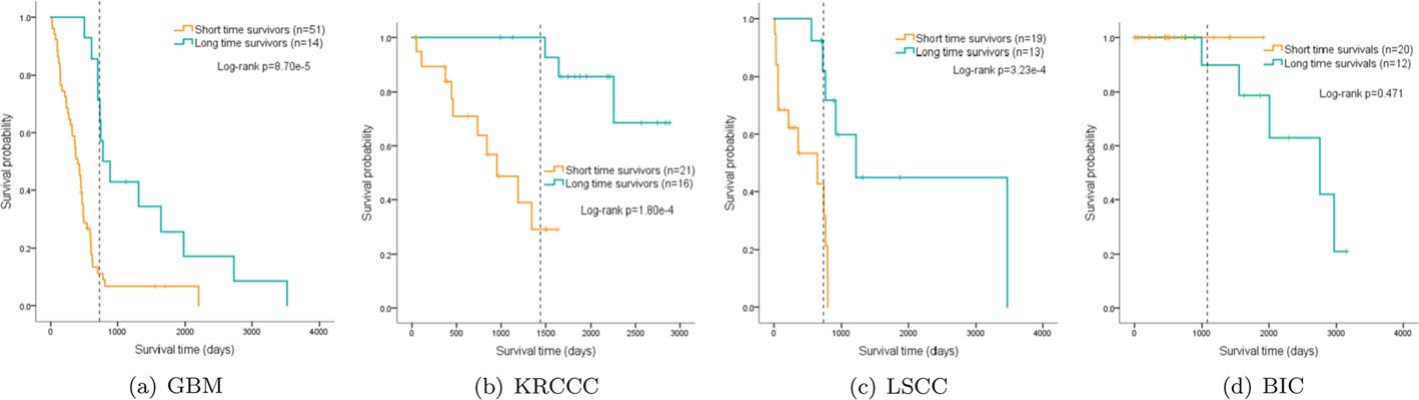
Kaplan–Meier curves of four cancers prognosis prediction. The dotted line in KM curve represents the median cut-off of two survivors

Survival analysis expresses a statistical method considering both results and survival time. Figure 8 shows the confusion matrixes of test sets on four cancers. The Kaplan-Meier (KM) survival curves are drawn in Fig. 9, and their P values are calculated according to the curves. For GBM, KRCCC and LSCC, there are significant differences between high-risk and low-risk patients (*p* values are $8.70 \times {10^{ - 5}}$, $1.8 \times {10^{ - 4}}$, $3.23 \times {10^{ - 4}}$, respectively), while for BIC, the difference is not significant ($p=0.471$). The *p* values for GBM, LSCC, KRCCC and BIC rise significantly when the censored data ratio rises from 0.077 for GBM to 0.875 for BIC. The reason is that the model can hardly learn well by primarily using the censored data.

### Effect of layer-by-layer constraints for strengthening feature representation ability

**Fig. 10:**
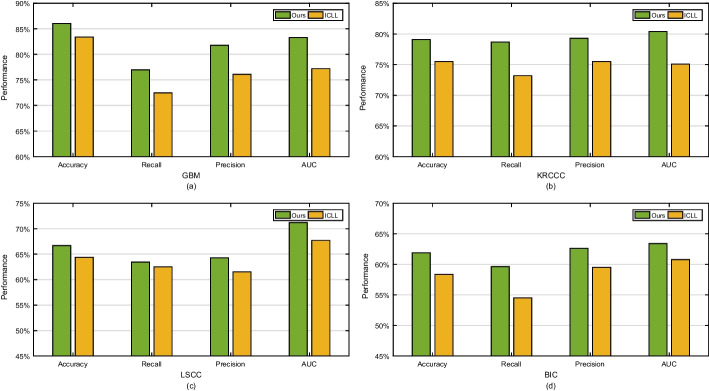
Prediction performance comparison between ICLL and ours

To investigate the effect of layer-by-layer constraints in our approach, we construct the compared backbone by *i*mposing *c*onstraints only on the *l*ast *l*ayer of deep learning network and denote it as ICLL. Figure 10 reports the comparison results of ICLL versus ours. Overall, our approach performs better than ICLL on all datasets in terms of accuracy, precision and AUC. The average performance improvements are 5.00%, 4.75%, 2.75% and 7.50% on GBM, KRCCC, LSCC and BIC datasets respectively, which indicates the effectiveness of imposing distance and orthogonal constraints layer-by-layer.

There are two reasons that the proposed approach is superior to ICLL that only imposes the constraints on the last layer of deep learning network: (i) layer-by-layer imposing constraints learns the shared and specific features multiple times, which can obtain better consensus and complementary feature representation than ICLL that learns shared and specific features only one time; (ii) layer-by-layer constraints are employed on each layer of deep learning networks, which can avoid learning networks falling into local optimal solution and can learn robust representations.

## Conclusion

Accurate prediction of survival time of cancers is significant, which can help clinicians make appropriate therapeutic schemes. State-of-the-art works show that integrating multi-type genetic data may be an effective way to deal with data heterogeneity, but they cannot provide a rational and feature representation for multi-type genetic data. To this end, we propose a deep learning approach which can learn the consensus and complementary information between multi-type genetic data at the feature level. It explicitly represents each type of genetic data as the shared and specific features to strengthen the interpretability. Sufficient experiments verify that the our approach can significantly improve the cancer survival prediction performance as compared with related works. In summary, our work provides an effective deep learning method to overcome data heterogeneity in cancer survival prediction.

## Data Availability

The datasets generated and analysed during the current study are available with http://compbio.cs.toronto.edu/SNF/.
